# Understanding the extracellular vesicle surface for clinical molecular biology

**DOI:** 10.1002/jev2.12260

**Published:** 2022-10-14

**Authors:** Susannah Hallal, Ágota Tűzesi, Georges E. Grau, Michael E. Buckland, Kimberley L. Alexander

**Affiliations:** ^1^ Neurosurgery Department Chris O'Brien Lifehouse Camperdown NSW Australia; ^2^ Brainstorm Brain Cancer Research, Brain and Mind Centre The University of Sydney NSW Australia; ^3^ Neuropathology Department Royal Prince Alfred Hospital Camperdown NSW Australia; ^4^ School of Medical Sciences Faculty of Medicine & Health The University of Sydney Camperdown NSW Australia

## Abstract

Extracellular vesicles (EVs) are lipid‐membrane enclosed nanoparticles that play significant roles in health and disease. EVs are abundant in body fluids and carry an array of molecules (proteins, lipids, nucleic acids and glycans) that reflect the identity and activity of their cell‐of‐origin. While the advent of high throughput omics technologies has allowed in‐depth characterisation of EV compositions, how these molecular species are spatially distributed within EV structures is not well appreciated. This is particularly true of the EV surface where a plethora of molecules are reported to be both integral and peripherally associated to the EV membrane. This coronal layer or ‘atmosphere’ that surrounds the EV membrane contributes to a large, highly interactive and dynamic surface area that is responsible for facilitating EV interactions with the extracellular environment. The EV coronal layer harbours surface molecules that reflect the identity of parent cells, which is likely a highly valuable property in the context of diagnostic liquid biopsies. In this review, we describe the current understanding of the mechanical, electrostatic and molecular properties of the EV surface that offer significant biomarker potential and contribute to a highly dynamic interactome.

## 1 INTRODUCTION

Extracellular vesicles (EVs) are phospholipid‐bound secreted nanoparticles that carry a complement of lipids, DNA, RNA, protein, metabolites, and glycans that reflect the identity and molecular state of their cell‐of‐origin (Yanez‐Mo et al., 2015). EVs are important mediators of intercellular communication, facilitating the secure delivery of molecular signals through the extracellular milieu (Morelli, 2017). EVs play integral roles in healthy (Jiang et al., 2021) and pathological states, including in cancer biology (Chistiakov & Chekhonin, 2014; Nakano et al., 2015), by contributing to the preparation of pre‐metastatic niches (Hoshino et al., 2015; Kaplan et al., 2005), stimulating cell proliferation and angiogenesis (Skog et al., 2008), and responding to environmental stimuli (Kucharzewska et al., 2013). Relative to cells, EVs carry significantly smaller molecular loads, yet they elicit powerful functional effects in recipient cells, implicating highly efficient and specific EV to cell recognition and uptake mechanisms (Margolis & Sadovsky, 2019).

EV surface chemistry is understood to have immense biological significance for regulating EV homing, targeting and uptake by recipient cells (Dang et al., 2020; Garofalo et al., 2019; Hoshino et al., 2015; Rana et al., 2011) and as biological effectors (Ipinmoroti, & Matthews, 2020; Reategui et al., 2018). The EV surface typically displays unique arrangements of molecules derived from parent cells and acquired through electrostatic interactions with extracellular molecules. Collectively, these molecules contribute to an exofacial coronal layer that surrounds the EV membrane and contributes to the molecular, physical and electrostatic properties of EVs. This coronal layer regulates EV intercellular interactions, mobility and biodistribution, and often contributes multiple key features for EV identification, classification and affinity isolation. In pathological conditions, EVs from aberrant cells are often secreted in large quantities into body fluids and possess properties that reflect specific disease states (Freeman et al., 2018; Reategui et al., 2018). As such, the EV surface likely comprises assets useful for the targeted capture of disease‐specific EV populations from body fluids and allow more sensitive assessments of cargo‐ed biomarkers. In this review, we describe the current understanding of EV surface chemistry and provide an overview of the EV surface molecular composition, the mechanical, hydrodynamic and electrostatic properties. Further, we highlight important EV surface features that facilitate intercellular communication and may enhance the utility of EVs as diagnostic biomarkers in clinical molecular biology.

## 2 EXTRACELLULAR VESICLE (EV) SUB‐TYPES, CLASSIFICATION AND NOMENCLATURE

While precise EV classification remains extraordinarily difficult, with no consensus for distinguishing physical or biochemical features of different sub‐types, the MISEV2018 guidelines support the use of the umbrella term ‘EV’, along with descriptions of physical traits (i.e., size and density), biochemical composition (e.g., surface protein and lipid composition) and cellular condition/cell‐of‐origin (e.g. podocyte EVs and hypoxic EVs) (Théry et al., 2018). Where EV identities cannot be thoroughly established, terms such as ‘extracellular particle’ are encouraged (Théry et al., 2018). Conventionally, EVs encompass three main classes of secreted vesicles that vary in size, content, origin and biogenesis; endosome‐derived ‘exosomes’ (30–100 nm), plasma membrane‐derived ‘microparticles’ (100–1000 nm; also known as ectosomes, shedding vesicles or microvesicles) and apoptotic cell‐derived apoptotic bodies (200–5000 nm) (Akers et al., 2013; Pavlyukov et al., 2018). Microparticles form by outward budding of the plasma membrane, while exosomes are secreted by plasma membrane fusion with multi‐vesicular bodies (MVBs) that form by invagination of late endosomal membranes into intraluminal vesicles (ILVs) (Akers et al., 2013). The remarkable diversity in EV size, morphology and cargo, however, challenges this conventional EV classification system.

Within EV isolates, atypically large EV sub‐types have been documented, including large oncosomes (1–10 μm) that are released by cancer cells and carry oncogenic material (Di Vizio et al., 2012; Minciacchi et al., 2015; Vagner et al., 2018; Zijlstra & Di Vizio, 2018), exophers (approximately 4 μm) that contain protein aggregates and organelles (Melentijevic et al., 2017), and migrasomes (>1 μm) that are released during migracytosis, a cell migration‐dependent mechanism of vesicular secretion (Ma et al., 2015). In addition, smaller particulates, known as exomeres, have been identified within EV isolates through the employment of asymmetric flow field‐flow fractionation (Zhang et al., 2018). Exomeres are small non‐vesicular populations (<50 nm) that are structurally, functionally and biochemically distinct to exosomes. Their exact composition is yet to be determined, yet evidence shows that they display unique protein, N‐glycosylation, lipid, RNA and DNA profiles, and are aggregates of molecules, rather than lipid membrane‐enclosed vesicles (Zhang et al., 2018). Exomeres are also enriched in proteins involved in metabolism and display distinct biodistribution patterns that are not typical for exosomes. Recently, a population of morphologically and molecularly distinct extracellular nanoparticles, termed ‘supermeres’, were identified in the supernatant of exomeres. Supermeres are rich in glycolytic enzymes, carry higher quantities of extracellular RNA and display a high efficiency of cellular uptake compared to small‐EVs and exomeres (Zhang et al., 2021). In this review, we use the term ‘EV’ to encompass studies of small (<200 nm) and large (>200 nm) extracellular membrane‐bound particle sub‐types.

## 3 MOLECULAR COMPOSITION OF THE EV SURFACE

The EV surface is surrounded by a coronal layer that encompasses an array of molecules (lipids, proteins, nucleic acid and glycans) that are integral and peripheral to the EV membrane. Typically, EV surface molecules are canonical to EVs and the cell‐of‐origin and acquired from the extracellular environment. The EV surface molecular repertoire is, therefore, influenced by EV sub‐type, host cell identity and molecular state and EV biodistribution patterns (Buzás et al., 2018). The EV surface expands the signalling capacity of EVs, allowing them to directly target and elicit functional changes in recipient cells, with and without membrane fusion, and mediate diverse intercellular signalling functions (Buzás et al., 2018; Sung & Weaver, 2017). Also resident to the EV surface are biomarkers that allow the molecular classification of diseases, EV sub‐typing and affinity isolation of specific EV populations (Tauro et al., 2013). The molecular construct of the EV surface is explored below and a summary is provided in Table 1.

**TABLE 1:** EV surface molecules identified through high‐throughput molecular profiling

EV surface localisation	Protein	Profiling method	EV source	Reference
*Integral to membrane*	ADGRG1, BCAM, BST2, CADM1, CADM4, CD109, CD44, CD46, CD47, CD70, CD81, CD9, CEACAM1, CEMIP2, CPD, DCBLD2, ECE1, ESYT2, FAM3C, GPC4, HSD17B12, IGF1R, IGF2R, IGSF3, IGSF8, ITGA5, ITGAV, ITGB5, ITM2B, L1CAM, LDLR, LNPEP, MCAM, MICA, MMP14, NCAM1, NECTIN2, NRP1, PLAUR, PLD3, PLEKHB2, PLP2, PODXL, PRNP, PTGFRN, SDK1, SERINC1, SLC7A6, SPINT1, ST14, SYNGR2, TFRC, TMEM87A, TSPAN1, TSPAN14, ULBP3, VAMP7, ZDHHC20	LC‐MS/MS of membrane proteins isolated by sodium carbonate extraction and phase separations. Transmembrane proteins predicted with web‐based membrane topology and signal peptide database (TOPCONS), and peripheral proteins predicted with UniProt database. Surface‐accessible proteins identified by Proteinase K cleavage off the EV surface.	Human colorectal cancer cell line SW480	(Xu et al., 2019)
*Peripheral to membrane*	ARCN1, CAPNS1, CDK5, CLU, DCXR, DNM1L, EIF3L, EPRS, FKBP1A, GANAB, LGALS3BP, RACK1, SEC23B, USO1			
*Surface accessible* ^a^	RNA binding proteins: AIMP2, DDX17, DDX6, DHX9, EIF2S1, EIF3A, EIF3B, EIF3L, EIF4G2, HNRNPC, HNRNPU, RPL6, RPL7, RPL9, SNRPD1, TSN, XPO7, XRCC6 DNA binding proteins: XRCC5, DHX9 Enzymes: ATP6V1E1, BLMH, CBR3, CDK2, CSNK1G2, DERA, GGT5, HTATIP2, MDH2, NAA50, PFAS, PKM, PKN2, PPP2R2A, PRMT5, PTP4A1, SHMT2, SOD1, TTLL12, UCHL3, UMPS, USP5, USP9X Other proteins: ACTC1, ACTL6A, ANP32B, APOE, ARF5, CHORDC1, DLG3, ECPAS, GNG12, GNG4, MELTF, MEMO1, PIP, PLEKHN1, PSMD10, RAB6B, RAB8B, RCC2, SH2D4A, SLC39A14, STX16, NPEPL1, TF, TGFBI, TMEM106B, TRIM28, TUBB8, UBL3			
*Integral to membrane*	Inside‐out membrane topology: SCAMP3, SLC12A6, SLC2A3, SPN Conventional membrane topology: ACSL4, BCAM, BSG, CD81, CD84, CD99, HEG1, HLA‐B, HLA‐G, ICAM1, IGSF3, IGSF8, ITGA6, KIT, LMAN2, MAOA, PLXNA4, RAB10, SDC4, SEMA7A	EV surface proteins cleaved off by Proteinase K and LC‐MS/MS. Subtractive proteome analysis to determine surface and luminal EV proteins. Verification of EV surface proteome by LC‐MS/MS of Trypsin‐Lys‐C proteolytically digested and biotinylated EV surface proteins. Identified surface proteins annotated as membrane or non‐membrane. Membrane proteins topology determined by proteolytic assessments.	Human mast cell line HMC‐1	(Cvjetkovic et al., 2016)
*Peripheral to membrane*	AHCYL1, DHRS2, GAPDH, GFPT1, KIF14, NCL, PGAM1, PPP1CB, PRKCB, RACGAP1, RPL8, SERBP1, STUB1, YWHAQ			
*Surface accessible* ^a^	Nascent‐EV surface proteins: ACTB, AHSG, ALB, ALDOA, ANPEP, ANXA2, ANXA4, ANXA5, ANXA6, ATP1A1, BASP1, BSG, GNAI2, CA2, CAP1, CD44, CD70, CFL1, CORO1A, CTSG, EZR, FCER1G, GAPDH, GC, GNAI3, GNB1, GNB2, GNB4, GSN, MSN, MYL6, ENO1, HLA‐A, HLA‐C, HSPA8, HSP90AA1, HSP90AB1, ITGAL, ITGB1, ITGB2, LDHA, LDHB, LGALS1, LYN, MYH9, MYO1G, NCAM2, PECAM1, PFN1, PGK1, PKM, PPIA, PRDX1, PTPRC/CD45, RAP1A, RAP1B, SLC3A2, SPN, TUBA1A, TUBB, TUBB4B, YWHAB, YWHAE, YWHAG, YWHAQ, YWHAZ EV surface coronal layer proteins acquired from human plasma: A1BG, A2M, ACTG1, AMBP, APOA1, APOA4, APOB, APOC3, APOD, APOE, APOH, AZGP1, C3, C4B, CFB, CFH, CFHR1, CP, CLU, EEF1A1, F2,FGA, FGB, FGG, HBA1, HPX, HP, HPR, HRG, IGHA1, IGHG1, IGHG2, IGHG3, IGHG4, IGHM, IGK, IGKC, IGKV3‐20, IGKV3D‐20, IGLC3, ITIH1, ITIH2, ITIH4, KNG1, LBP, LPA, ORM1, ORM2, PLG, PON1, RBP4, SERPINA1, SERPINA3, SERPINC1, SERPINF2, TF, TTR, SERPIND1, VTN	EVs isolated by density centrifugation, isopycnic ultracentrifugation, or size exclusion chromatography. Nascent EV proteins were assessed by LC‐MS/MS. Nascent EVs incubated with platelet/EV‐depleted plasma to test which proteins are acquired within the coronal network. EVs were re‐isolated and proteins assessed by LC‐MS/MS	Human THP1 cell	(Tóth et al., 2021)
*Surface accessible* ^a^	Cytokines: Calg‐A, Eotaxin, GM‐CSF, GRO‐α, IL‐1α, IL‐1β, IL‐2, IL‐6, IL‐7, IL‐8, IL‐12, IL‐13, IL‐16, IL‐17, IL‐18, IL‐21, IL‐22, INF‐γ, IP‐10, I‐TAC, MCP‐1, M‐CSF, MIG, MIP‐1α, MIP‐1β, MIP‐3α, RANTES, TNF‐α, TGF‐β	Surface cytokines measured using a multiplex bead‐based immunoassay, before and after detergent treatment (1% Triton X). Surface‐associated cytokines verified with trypsinisation. Cytokines reported display pre‐dominant EV surface expression (>50%) of total EV‐associated cytokines.	EVs from human placental villous explants, amnion explants, tonsillar explants, cervical explants, plasma, amniotic fluid, T cells and monocytes.	(Fitzgerald et al., 2018)
*Surface accessible* ^a^	N‐Glycoproteins: FN1, CD44, OLFM4, CD47, CD157, CD11b, CD97, LGALS3BP, CD39, CD18, CD321, MPO, THBS1, CD82, KRT77, FGB, CD177, MCEMP1, TM9SF3, CD41, LIPG	Oxidation of EV surface N‐glycoproteins with oxy‐amino alkylation method, followed by LC‐MS/MS analysis of biotinylated oxidated peptides.	Mouse myeloid‐derived suppressor cells	(Chauhan et al., 2017)
*Surface accessible* ^a^	N‐Glycoproteins: AHSG	Mass spectrometry, and antibody inhibition during functional assays of plasma‐EVs targeting human mesenchymal stromal cells.	Human plasma	(Wu et al., 2019)
*Surface accessible* ^a^	O‐glycoproteins: MUC1, MUC4, MUC16	Mass spectrometry analysis of EVs derived from human tracheobronchial epithelial cells. Surface‐bound mucin proteins verified by flow cytometry and immuno‐electron microscopy.	Human airway epithelial cells	(Kesimer et al., 2009)

**EV surface localisation**	**Lipids**			**Profiling method**	**Source**	**Reference**
*Membrane*	**Large‐EVs (100–200‐nm mean size)**	**Small‐EVs (<100‐nm mean size)**	Small and large EVs separated by differential centrifugation, assessed by LC‐MS/MS lipidomic analysis. Data expressed as the percentage of the total lipid content. Membrane cholesterol content measured through a colorimetric assay	Murine differentiated adipocyte cell 3T3‐L1	(Durcin et al., 2017)
	***Phospholipids***					
	Phosphatidylcholine	58%	58%			
	Phosphatidylethanolamine	4.25%	7%			
	Phosphatidylinositol	4.5%	4%			
	Phosphatidylserine	0.7%	2%			
	Lysophosphatidylcholine	6.8%	5%			
	***Sphingolipids***					
	Ceramide	0.57%	0.35%			
	Glycosylceramides	0.028%	0.025%			
	Sphingomyelin	24.2%	22.9%			
	***Glycerolipids***					
	Diacylglycerols	0.9%	1.4%			
	***Cholesterol***	2‐fold enrichment in small EVs				

**EV surface localisation**	**Nucleic acid**	**Profiling method**	**Source**	**Reference**
*Peripheral*	let‐7a‐5p	Proteinase K digestion of RNA binding proteins and RNase digestion of their associated RNA at the surface of intact SW480‐EVs.	Human colorectal cancer cell‐line SW480	(Xu et al., 2019)
*Peripheral*	Chromosomal DNA sequences (GAPDH and p53) and mitochondrial DNA sequences (mitochondrial (mt) control gene and 12S RNA (RNR1))	DNA sequences amplified by PCR and analysed by agarose gel electrophoresis.	Human lymphoma cell line	(Németh et al., 2017)
*Peripheral*	Chromatin	Enzymatic digestion of microparticle surface chromatin.	Human plasma	(Sisirak et al., 2016)

^a^ Molecules identified at the EV surface are annotated as ‘surface accessible’ if their relative localisation to the EV membrane (i.e., integral or peripheral) has not been confidently defined in the respective molecular profiling study.

### 3.1 EV membrane lipids

Lipids are the fundamental structural components of the EV membrane, and there is a growing interest in EV lipids as predictive biomarkers of cancer progression (Brzozowski et al., 2018), neurodegenerative diseases (Wang et al., 2021) and diabetes (Donoso‐Quezada et al., 2021). Distinct lipid signatures are characteristic to several cancer types (Marien et al., 2015; Menendez & Lupu, 2007; Rysman et al., 2010), non‐fatty liver disease, obesity and virus‐infected cells (Ameer et al., 2014; Smith et al., 2020), and it is likely that lipidomic changes are mirrored in the EV membrane across different physiological states (Llorente et al., 2013). Lipids constitute approximately 50% of the mass of eukaryotic membranes (Alberts & J.A., 2002) and include glycerophospholipids such as phosphatidylcholine (PC), phosphatidylethanolamine (PE), phosphatidylserine (PS), phosphatidylinositol (PI) and phosphatidic acid (PA), as well as, sphingolipids (sphingomyelin, glycosphingolipids and ceramides), cholesterol and glycolipids (Alberts & J.A., 2002; van Meer et al., 2008). Glycerophospholipids contribute to the structural framework of membranes as their amphipathic nature leads to spontaneous self‐assembly (van Meer et al., 2008) and the formation of highly fluid and energetically favourable lipid membrane compartments (Alberts & Johnson, 2002).

**FIGURE 1:**
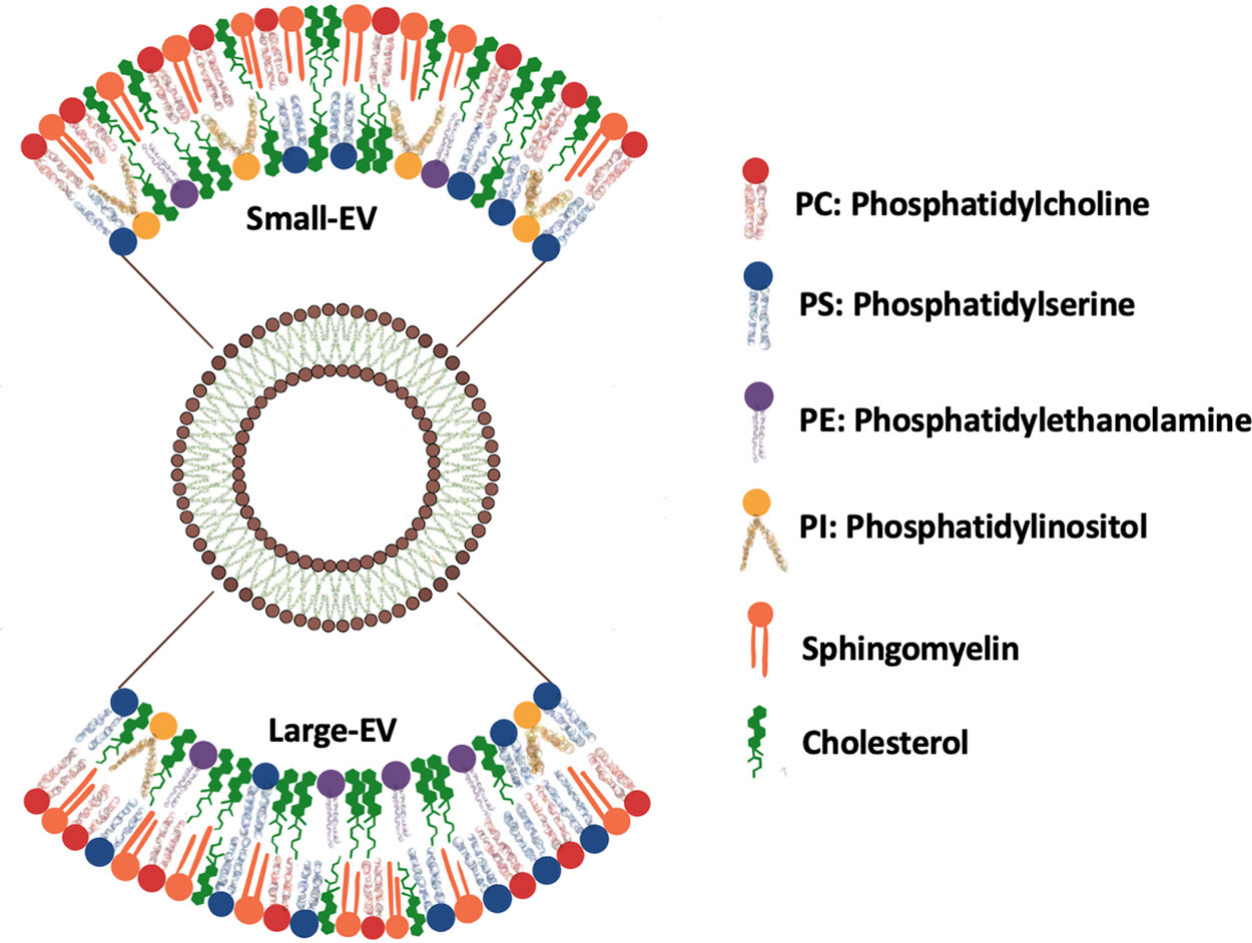
The main lipid components and their general distribution across the two leaflets of the membrane bilayer of small and large‐EV sub‐types. EV membranes are typically comprised of phosphatidylcholine (PC) and sphingomyelin in the outer leaflet. PC and phosphatidylserine (PS) are phospholipids with a cylindrical shape and contribute to the formation of flat bilayer sheets. Approximately 60% of the total EV‐associated PS is externalised to the outer leaflet in larger EV sub‐types, while PS is localised on the inner leaflet of small‐EV sub‐types. Cholesterol is distributed across the two leaflets and is enriched two‐fold in the membranes of small‐EVs compared to larger‐EVs. Phosphatidylethanolamine (PE) is also enriched two‐fold in smaller‐EVs compared to larger‐EV sub‐types and is a conical lipid that exerts a positive curvature to membranes by encouraging the phospholipid heads to pack closer together during membrane bending. Phosphatidylinositol (PI) has an inverted conical shape and exerts a negative curvature to membrane bilayers.

Like cellular membranes, EV membranes include a range of glycerophospholipids (PC, PE, PI and PS), sphingolipids (sphingomyelin, ceramides and glycosphingolipids, glycosylceramides), glycerolipids (diacylglycerols), cholesterol and glycolipids. Comprehensive lipidomic mass spectrometry analyses of EVs derived from adipocytes (Durcin et al., 2017), and metastatic prostate cancer (Llorente et al., 2013) and human B cell (Wubbolts et al., 2003) cell lines, show that EV membranes are mainly comprised of PC (15‐58%) and cholesterol (approximately 40%), followed by sphingolipids (8%–24%), PS (2%–15%), proportionally minor levels of PI (0.1%–4%) and PE (1%–7%), and trace amounts of ceramide, glycosylceramide and diaglycerols (≤1%) (Table 1 and Figure 1) (Durcin et al., 2017; Katarina et al., 2008; Llorente et al., 2013; Wubbolts et al., 2003). EV membranes display unique lipid expression and distribution patterns compared to cellular membranes (Laulagnier et al., 2004; Llorente et al., 2013) suggesting that lipids are integrated into the EV membrane through highly regulated processes, and that EV membranes are not simply remnants of cellular membranes. For example, in a study comparing EV and plasma membranes of prostate cancer PC‐3 cells, EV membranes contained 2–3 times more cholesterol, sphingomyelin, glycosphingolipids and PS, and half the content of PC (Llorente et al., 2013). While the plasma membrane and endocytic membranes display diverse lipid composition and distribution (Bissig & Gruenberg, 2013; Urade et al., 1988), it is not well understood how EV biogenetic pathways influence EV lipid composition, and how the lipid membrane of EV sub‐types compares to the membranes of their respective biogenetic pathways.

Generally, lipid membranes display an asymmetric distribution of the lipid classes across the two leaflets, with sphingolipids and PC typically in the outer leaflet, and other lipid classes distributed across the inner leaflet (Skotland et al., 2019). Across EV membranes, sphingomyelin and PC are typically found on the outer leaflet, while PE, ether‐linked PE (PE‐O), and PI are found on the inner leaflet (Figure 1) (Skotland et al., 2020). Lipid orientation and distribution patterns across the EV membrane influence protein aggregation, membrane curvature and EV shape, which is critical to EV secretion morphology and signalling (van Meer et al., 2008), and varies significantly across EV sub‐types (Théry et al., 2018). Comprehensive lipidomic analyses of adipocyte‐EVs and infrared spectroscopy analyses of EVs derived from Jurkat T‐cells, prostate cancer cells and melanoma cells have shown that small (<100 nm), intermediate (100–450 nm) and large (>450 nm) EV sub‐types display unique lipid profiles (Durcin et al., 2017; Mihály et al., 2017; Paolini et al., 2020). Relative to the plasma membrane of their parent Jurkat cells, large apoptotic bodies are similar in composition, while intermediately sized microvesicles share high levels of phospholipids, and small exosomes are enriched in cholesterol, sphingomyelin and ceramide (Mihály et al., 2017).

Mass spectrometry lipidomic analyses of large (100–200 nm) and small‐EV (<100 nm) sub‐types from adipocyte 3T3‐L1 cells showed similar concentrations of phospholipids and sphingolipids, while cholesterol was enriched in small‐EVs by 2‐fold (Table 1) (Durcin et al., 2017). The membranes of the large‐EVs were also characterised by high levels of externalised PS with flow cytometric assessments (approximately 60% of total EV‐associated PS) (Figure 1) (Durcin et al., 2017). In the cell membrane, PS is typically located on the inner membrane leaflet, and enzymatic remodelling translocates PS to the outer leaflet of large‐EVs such as apoptotic bodies and microvesicles (Heijnen et al., 1999; Skotland et al., 2020), where it is externalised and exposed to the extravesicular environment and functions as an ‘eat me’ signal for macrophages (Matsumoto et al., 2017; Segawa & Nagata, 2015). Externalised PS also mediates EV interactions with receptors of recipient cells to facilitate cellular uptake (Véron et al., 2005). For example, externalised PS on EVs forms a bridge with MFGE8, which can then bind to integrins on the surface of dendritic cells to amplify the immune response (Véron et al., 2005). Externalised PS also exerts a pro‐coagulant function for EVs as it contributes to a negatively charged anionic EV surface for the binding of coagulation factors such as VIII, Va and Xa (Heijnen et al., 1999). In contrast, smaller‐EVs, such as exosomes, typically exhibit less PS in the outer leaflet (Durcin et al., 2017), which may contribute to exosome evasion of immunological clearance from the circulation, thereby extending their circulatory half‐life. Exosome membranes also contain a high percentage of cholesterol in both the inner and outer leaflets (Vidal et al., 1989), where it functions to maintain membrane dynamics, increase membrane lipid order and prevent ion leakage (Skotland et al., 2020).

In the context of cancer, differences in EV lipid content can distinguish tumour phenotypes from non‐tumour phenotypes and predict cancer progression. It was shown that EVs from seven tumour‐derived cell lines of epithelial and myeloid origin contain less cholesterol and higher phospholipid content than EVs from non‐cancerous cell lines (Smith et al., 2015), while EVs from tumorigenic and metastatic prostate cells display a higher abundance of ceramide relative to EVs from normal prostate cells (Brzozowski et al., 2018). In addition, tumour‐derived EVs display lower levels of fatty acids, glycerolipids and prenol lipids, and higher levels of sphingolipids and glycerophospholipids (Brzozowski et al., 2018). Higher levels of surface‐exposed PS have also been observed in EVs from hypoxic cells (Wei et al., 2016) and tumour cells (Lima et al., 2009; Matsumura et al., 2019; Muhsin‐Sharafaldine et al., 2016), which may serve as a promising marker for detecting early stage malignancy and tumour progression (Sharma et al., 2017); affinity‐based methods that target and capture EV surface PS for monitoring EV levels in patient blood are under development (Nakai et al., 2016).

Currently, there are few lipidomic studies of EVs in health and disease, and the EV lipidome is not yet adequately characterised. A major hurdle to in‐depth lipidomic profiling relates to the technical challenge of isolating pure EV preparations free from lipoprotein contamination. It is suggested that more than 70% of EV isolates from complex body fluids, such as plasma, are not in fact EVs, but highly abundant lipoprotein and chylomicron contaminants that exhibit similar buoyant densities and sizes to EVs (Sódar et al., 2016; Yuana et al., 2014). Typically, the size range of chylomicrons are 75–1200 nm, very‐low‐density lipoproteins (VLDL) are 30–80 nm, and low‐, intermediate‐ and high‐density lipoproteins (LDL, IDL, HDL) are 5–35 nm (Karimi et al., 2018). Together, different lipoprotein species outnumber EVs in the plasma by six orders of magnitude (Johnsen et al., 2019). In addition, HDL has a similar density to EVs (Johnsen et al., 2019), and the overlapping density and sizes of lipoparticles with EVs makes isolating pure EV populations very challenging. Lipoproteins commonly interact with the EV surface, co‐isolate with EV preparations (Sódar et al., 2016; Yuana et al., 2014), and affect the accuracy of EV lipidomic assessments and EV particle counting (Skotland et al., 2017).

**FIGURE 2:**
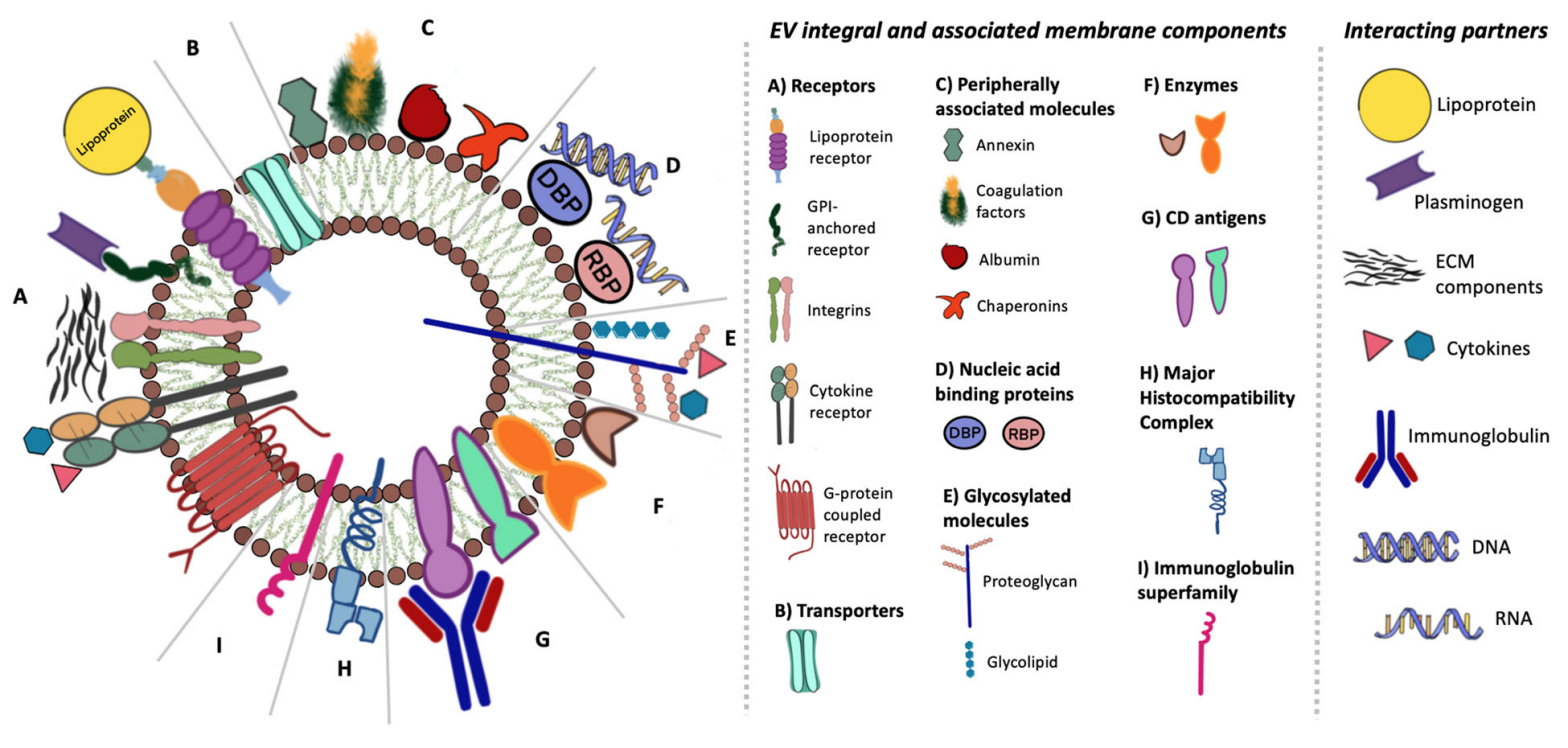
Molecular components that comprise the EV surface. Molecules that are integral to the EV membrane include receptors (G‐protein‐coupled receptors, cytokine receptors, integrins, GPI‐anchored receptors and lipoprotein receptors), transporters, immunoglobulin superfamily proteins, major histocompatabilty complex, glycoproteins, enzymes and CD antigens. Peripheral molecules also contribute to the EV surface molecular repertoire by binding to the integral proteins or interacting with the EV lipid membrane. Molecules that interact with integral EV membrane proteins include cytokines, extracellular matrix components, plasminogen, lipoproteins and immunoglobulins. Molecules that have been shown to interact with the EV membrane include enzymes, DNA/RNA binding proteins, DNA, RNA, annexin, albumin and components of the coagulation cascade.

Lipoprotein contamination in human blood EV isolates may be reduced using a combination of isopycnic gradients and size exclusion chromatography (Karimi et al., 2018); size exclusion chromatography allows small‐EV sub‐types to be separated from smaller lipoproteins, while isopycnic gradients enrich EVs from chylomicrons and VLDL that have distinct densities (Karimi et al., 2018). Depletions of lipoproteins via antibody‐mediated (Mørk et al., 2017) and lipoprotein apheresis (Connolly et al., 2014) have also been trialled, yet lead to overall reduced EV yields possibly due to lipoprotein interactions with lipoprotein receptors at the EV surface (Table 1 and Figure 2). More recent advances, such as integrated dual mode chromatography, have allowed the removal of lipoprotein contaminants from EV preparations without compromising EV yields. Dual mode chromatography integrates size exclusion chromatography to remove HDLs that are smaller than EVs, and strong cation exchange to remove positively charged VLDLs from negatively charged EVs (Van Deun et al., 2020). Future standardisation and improvement of EV isolation and profiling technologies will improve EV‐based lipidomic characterisations for biomarker discovery and allow for more holistic and in‐depth characterisations of the EV surface molecular repertoire.

### 3.2 EV surface proteome

The EV surface comprises an array of proteins from a range of functional classes, that are both integral and peripheral to the EV membrane. Proteins that are intrinsic to the EV phospholipid membrane span up to 16 α‐helical transmembrane domains, while peripheral proteins interact with the EV surface through non‐covalent interactions with the integral membrane proteins or through electrostatic interactions with hydrophilic phospholipid head groups (Xu et al., 2019). Broadly, proteins at the EV surface include a range of tetraspanins (CD9, CD63, CD81) (Kowal et al., 2016), transport/fusion proteins (annexin, flotillin, RABs), antigen presentation proteins (major histocompatibility complex, MHC class I and II), integrins, cell adhesion proteins, receptors [growth factor, cytokine, signal transduction and lipoprotein receptors (Reategui et al., 2018; Théry et al., 2018), chaperonins (Radons & Multhoff, 2005), heparin‐binding proteins (Balaj et al., 2015), enzymes, coagulation factors, nucleic acid binding proteins and immunoglobulin superfamily proteins (Table 1 and Figure 2) (Cvjetkovic et al., 2016; Xu et al., 2019). EV surface proteins reflect the parent cell's identity and state, and the surface protein composition can differentiate EV sub‐types (Belov et al., 2016; Castillo et al., 2018).

The EV surface and encapsulated luminal proteomes are distinct; the EV surface is largely comprised of cytoplasmic (51.1%) proteins, and 2‐fold more nuclear proteins (16.8%) compared to the EV lumen (8.3%) (Cvjetkovic et al., 2016). EV surface proteins are enriched in functional pathways related to protein translation (eukaryotic initiation factors, tRNA synthetases, ribosomal proteins) and cellular and protein metabolism (proteasomes, mitogen‐activated protein kinases and protein kinase C), while the encased EV protein cargo is associated with protein localisation (ARFs, Rabs, Exortins, Importins) and transport (syntaxins, transportins and sorting nexins) (Cvjetkovic et al., 2016). It is postulated that EV surface proteins impart similar functions as their plasma membrane counterparts, especially if they retain the same membrane orientation. However, mass spectrometry of enzymatically shaved EV surface peptides from mast cells revealed that around one‐third of EV‐associated transmembrane and lipid‐anchored proteins display a reversed, non‐conventional membrane orientation (Cvjetkovic et al., 2016), which may impart greater diversity to EV functionality. In this study, four transmembrane proteins (SCAMP3, SLC12A6, SLC2A3 and SPN) were conclusively determined to display reverse topology at the EV surface (Table 1) (Cvjetkovic et al., 2016). While this study assessed the topology of EV‐associated transmembrane and lipid‐anchored proteins, accurate topological assessments of protein–protein interactions at the EV surface is challenging due to soluble proteins that commonly contaminate EV preparations and form transient interactions with the EV surface. Distinguishing co‐isolating protein contaminants from true EV surface proteins is important for understanding interacting proteins and their topology at the EV surface (Mellacheruvu et al., 2013).

Characterising EV surface protein topology is critically important to our understanding of EV‐mediated intercellular communication and for conceptualising clinically relevant EV surface biomarkers. However, the high percentage of transmembrane proteins with reverse orientation at the EV surface (Cvjetkovic et al., 2016) complicates EV surface profiling by conventional immuno‐labelling methods. Immunoassays such as immunogold electron microscopy (György et al., 2017), flow cytometry (Maia et al., 2020), antibody microarrays (Belov et al., 2016; Jørgensen et al., 2013), confocal immunofluorescence (Lai et al., 2015), super resolution microscopy (Buono et al., 2021) and surface plasmon resonance (SPR) biosensing (Grasso et al., 2015) have been pivotal for characterising and visualising EV surface molecular distribution, co‐localisation and targetability (Andrade et al., 2021; Buono et al., 2021; Tauro et al., 2013). Unfortunately, they provide a myopic, oversimplified view of the EV surface as they can only assess a few markers simultaneously and are generally predicated on the EV surface sharing the same protein composition and orientation as the cell surface (Garnier et al., 2013; Reategui et al., 2018). In the context of reverse orientation of EV surface proteins, immunoassays may contribute to inaccurate EV surface protein assessments as reverse protein orientation may conceal epitopes from immuno‐detection (Fitzgerald et al., 2018).

Furthermore, proteomic analyses of the EV surface is challenging due to the intrinsic hydrophobicity, heterogeneity and low relative abundance of membrane proteins that contribute to their under‐representation in global proteomic analyses (Xu et al., 2019). The complexity of the EV surface proteome is further compounded by dynamic protein interactions that change with time and spatial distribution. Recent technical advances have allowed successful profiling of the EV surface proteome by mass spectrometry. In conjunction with methods that preserve the proteomic features of the EV surface, such as phase separations (Xu et al., 2019), proteolytic digestions (Cvjetkovic et al., 2016; Xu et al., 2019), biotinylation (Castillo et al., 2018; Cvjetkovic et al., 2016) and cross‐linking (Wittig et al., 2021), mass spectrometry has allowed the identification of proteins that are integral and peripheral to the EV membrane (Table 1), and described their interactions, accessibility and topology at the EV surface (Cvjetkovic et al., 2016; Xu et al., 2019). Immunoassays have also been useful for enriching mass spectrometric assessments of EV surface profiles, identifying a range of membrane proteins, as well as cytokines and enzymes (Table 1), that could further guide novel strategies for targeted EV immune‐based clinical assays (Liu et al., 2015; Redzic et al., 2014; Wittig et al., 2021).

Cytokines, chemokines and angiogenic proteins are secreted into the extracellular environment in free‐form or associated with EVs (Fitzgerald et al., 2018; Skog et al., 2008). Across several biological systems, such as human placental, amnion, tonsillar, and cervical explants, amniotic fluid, plasma, T‐cells and monocytes, multiple cytokine species exhibit preferential expression in the soluble form (IL‐6, IL‐13, IP10, MCP‐1, MIP‐1α and MIP‐1β), while others are more likely to be associated with EVs, pre‐dominantly at the EV surface (IL‐8, IL‐17 and GRO‐α) or as internal cargo (IL‐2, IL‐4, IL‐10, IL‐12, IL‐15, IL‐16, IL‐18, IL‐21, IL22, IL‐33, Eotaxin, IP‐10, ITAC, M‐CSF, MIG, MIP‐3α, TGF‐β and TNF‐α) (Fitzgerald et al., 2018).

Interestingly, cytokine packaging and localisation within EVs appears to be associated with tightly regulated biological processes that are dependent on the parental cell's identity, molecular state, response to external stimuli and EV biogenesis (Fitzgerald et al., 2018). Using multiplexed bead assays, unique EV surface cytokine profiles have been observed for different biological systems, with IL‐13, IFN‐γ and M‐CSF uniquely expressed on cervical explant EVs, eotaxin on tonsillar explant EVs, and IP‐10 on placental‐EVs (Fitzgerald et al., 2018). The surface of cancer cell‐derived EVs are characterised by the presence of IL‐8, GROα, TIMP‐1 and VEGF (Ko et al., 2019). Intriguingly, the majority of the EV‐associated VEGF is distributed at the surface and VEGF splice variant isoforms display preferential localisation across EV sub‐types; VEGF_189_ is enriched on the surface of small‐EVs (<200 nm) (Ko et al., 2019), while VEGF_90K_ is exclusively expressed on the surface of large‐EVs (500–1000 nm) (Feng et al., 2017). This suggests that protein sorting and orientation at the EV surface is a highly regulated process that occurs during EV biogenesis, and only a minor portion of the EV surface cytokine repertoire are soluble proteins that have associated to the EV surface after secretion (Ko et al., 2019). Furthermore, external stimuli can modulate patterns of cytokine secretion and EV localisation. Following stimulation, activated monocytes secrete EVs with relatively higher levels of surface‐bound cytokines such as IL‐1β, IL‐18, GRO‐α, IP‐10, M‐CSF, MCP‐1 and MIP‐1α (Fitzgerald et al., 2018).

Cytokines associated with the EV surface are more stable compared to free soluble cytokines (Fitzgerald et al., 2018; Ko et al., 2019). For example, VEGF_189_ is protected from enzymatic degradation by its interaction with heparan sulphate proteoglycans (HSPGs) on the EV surface (Figure 2E) (Ko et al., 2019). In addition, the surface of tumour‐EVs allows VEGF_189_ to evade detection by VEGF antibody, bevacizumab, prolonging VEGF_189_ half‐life and contributing to therapeutic resistance (Ko et al., 2019). Similarly, GROα and IL‐8 also interact with HSPG, which may contribute to their observed expression and protection at the EV surface (Ko et al., 2019). Shielded from environmental degradation, these EV surface‐associated cytokines maintain their signalling competency, irrespective of cellular uptake (Fitzgerald et al., 2018). At the EV surface, cytokines such as VEGF, IL‐8, CXCL1, IL‐6 and TGF‐β, have well‐established functions in stimulating cell growth, proliferation, migration, angiogenesis and/or tube formation (Buzás et al., 2018; Fitzgerald et al., 2018; Treps et al., 2017) and in autoimmunity, synovial fibroblast EVs display significant surface expression of TNFα that can activate T‐cell Akt and NFκB pathways to impede cell apoptosis (Zhang et al., 2006). While EV targeting to recipient cells is generally promoted by sialic‐acid binding lectins, C‐type lectins (Black et al., 2016), lactadherin (Dasgupta et al., 2009), MHC I (Utsugi‐Kobukai et al., 2003) and II (Muntasell et al., 2007) receptors, transferrin receptors and tetraspanins (Calzolari et al., 2006), it is possible that alternative EV targeting mechanisms are also mediated through EV surface cytokines binding to cytokine receptors at the recipient cell surface (Barnes & Somerville, 2020).

Cytokine receptors are also expressed on the EV surface where they form peripheral interactions with soluble cytokines (Figures 2A, 2E) (Cossetti et al., 2014). For example, neural stem cells secrete EVs that express surface interferon gamma receptor 1, which forms a complex with soluble IFN‐γ and activates STAT1 signalling in target cells more efficiently than soluble IFN‐γ (Cossetti et al., 2014). Cancer‐derived EVs present TGF‐β at their surface as a fully functional complex with type III TFG‐β receptor (also known as heparan sulphate proteoglycan betaglycan) that elicits SMAD‐dependent signalling and induces cellular differentiation in target cells (Webber et al., 2010). It is likely that the interactions between surface cytokine receptors and their respective soluble cytokines are highly dynamic and dependent on environmental conditions (i.e. pH, viscosity and temperature) as well as EV biodistribution.

Multiple proteases and glycosidases also contribute to the EV surface repertoire, where they are enzymatically active and can degrade extracellular matrix (ECM) molecules, release growth factors and alter cell adhesion (Sanderson et al., 2019). These enzymes are hypothesised to be distributed at the EV surface during endocytic secretory pathway biogenesis as transmembrane proteins such as MT1‐MMP (Hakulinen et al., 2008), or bound to components of the EV membrane (e.g. heparinase binds to heparan sulphate chains from syndecan‐1 at the EV surface; Figures 2E, 2F) (Sanderson et al., 2019; Thompson et al., 2013). Various cell types secrete EVs with surface proteases, including metalloproteases, hyaluronidase, kinases, ATPase, synthases, transferases, hydrolases, phosphatases (Table 1), insulin‐degrading enzyme and glycosidases (i.e. sialidase neuraminidase (NEU3) and heparinase) (Sanderson et al., 2019). EV surface enzymes are potentially important in pathological processes, such as inflammation, neurodegeneration and tumour progression and invasion (Sanderson et al., 2019). Indeed, there are positive associations between the quantity of shed vesicles, the abundance of EV‐associated proteolytic enzymes, and tumour aggression (Ginestra et al., 1998).

### 3.3 EV surface glycoproteins and glycans

EV‐associated proteins harbour a range of post‐translational modifications (PTMs), including glycosylation, acetylation, phosphorylation, oxidation, methylation and ubiquitination. Of these, glycosylation is the most adequately characterised modification at the EV surface. Up to 85% of human extracellular proteins are glycosylated, a process by which glycans (approximately 10 monomeric carbohydrate units) are covalently attached to protein amino‐acid side chains in four ways: (1) N‐linked to asparagine, (2) O‐linked to serine, threonine or tyrosine, (3) C‐linked to tryptophan and (4) glypiation (Kailemia et al., 2017; Schjoldager et al., 2020). The process of glycosylation diversifies protein functionality, conferring important roles in protein folding, stability, transport and signalling (Schjoldager et al., 2020), and influences cellular growth, differentiation, adhesion and metastasis (Guo et al., 2007). Generally, glycosylation is targeted to specific proteins at the cell surface, enabling cell‐specific selection (Schjoldager et al., 2020). Glycosylation is also influenced by numerous cellular and environmental factors, with aberrant cell surface glycoprotein expression observed in tumour progression (Kailemia et al., 2017; Schjoldager et al., 2020). Similarly, the EV surface carries an assortment of resident glycoproteins that are representative of the surface of the cells that released them; EVs from myeloid‐derived suppressor cells share approximately 80% of N‐linked glycoproteins as their parent cells (17 of 21 surface proteins: Table 1) (Chauhan et al., 2017).

In plasma‐derived EVs, 46% of N‐linked glycosylated proteins have been annotated as EV proteins in ExoCarta (Sok Hwee Cheow et al., 2015), and the majority of glycosites on the EV surface are topologically assigned to extracellular regions (Chauhan et al., 2017). EV surface glycome investigations have employed lectin arrays for the selective capture and assessment of glycans surrounding intact EVs (Batista et al., 2011; Gerlach et al., 2013; Krishnamoorthy et al., 2009) and mass spectrometry for high‐throughput proteomic analyses (Chauhan et al., 2017). Relative to the plasma membrane, the EV surface is enriched in polylactosamine, α2‐6 linked sialic acid residues (Shimoda et al., 2017) and complex type N‐glycans (Costa et al., 2018). The EV surface also carries a small amount of high mannose structures (Costa et al., 2018), and is depleted in terminal blood group A and B carbohydrate antigens (Batista et al., 2011; Krishnamoorthy et al., 2009). These glycan features have been observed on EVs derived from carcinoma and melanoma cell‐lines (Batista et al., 2011), human adipose‐derived mesenchymal stem cells (Shimoda et al., 2017), human embryonic kidney cells, human and mouse glioma cells (Costa et al., 2018), breast milk (Batista et al., 2011) and urine (Gerlach et al., 2013).

Together, glycans heavily decorate the EV surface and impart a negative charge (Kesimer et al., 2009). Transmission electron microscopy (TEM) imaging shows that glycoproteins are filamentous and entangled structures at the EV surface, that contribute to a surrounding coronal layer of 10–30 nm in thickness (Kesimer et al., 2009). The filamentous structures on the surface of airway epithelial EVs are heavily O‐glycosylated mucins with a high molecular weight (MUC1, MUC4 and MUC16) (Kesimer & Gupta, 2015), that function to fortify EV structure (Kesimer et al., 2009) and contribute to EV hydrodynamic diameter (Kesimer & Gupta, 2015). Interestingly, EV surface glycans are also direct facilitators of cellular uptake and significantly influence recipient cell activity (Williams et al., 2019). The EV membrane is abundant with transmembrane glycosaminoglycan and proteoglycan species (Figure 2E) that mediate EV internalisation, including HSPGs, syndecans and glypicans (Christianson et al., 2013). Sialic acid residues enriched on EV membranes, also play important roles in EV uptake, and contribute to immunoglobulin‐type lectin receptor (siglecs) binding at the cell surface (Shimoda et al., 2017) and influence EV biodistribution patterns in vivo (Royo et al., 2019). On the surface of EVs derived from myeloid‐derived suppressor cells, N‐glycoproteins CD47 and THBS1, contribute to chemotaxis and migration in recipient cells (Chauhan et al., 2017). Similarly, α2‐HS glycoprotein at the surface of plasma‐EVs facilitates their uptake by mesenchymal stromal cells, leading to a reduction in endogenous pSmad 1/5/8 levels and inhibiting osteogenic differentiation (Wu et al., 2019). It is unclear whether glycan‐mediated cellular uptake of EVs is exclusively based on glycan–receptor interactions at the cell surface or whether it is also a function of charge and electrostatic effects (Williams et al., 2019).

As EV glycosylation profiles can be readily distinguished from other highly glycosylated proteins that commonly co‐isolate with EV preparations, such as Tamm–Horsfall Protein (THP), EV surface glycan profiles show great promise as diagnostic and predictive biomarkers (Gerlach et al., 2013; Nyalwidhe et al., 2013). Differential N‐linked glycosylation profiles have been observed for urinary EVs from patients with polycystic kidney disease (Gerlach et al., 2013) and plasma‐EVs from breast cancer patients (Chen et al., 2018). Similarly, O‐glycosylation modification levels are significantly higher in EVs from metastatic colorectal cancer cells (Chaiyawat et al., 2016). EVs are also enriched in fucosylated glycan structures that have been linked with cancer progression and inflammation (Saraswat et al., 2015). In breast cancer, the highly glycosylated form of ECM metalloproteinase inducer (EMMPRIN) is preferentially expressed on larger‐EV sub‐types, and is also a marker for tumour invasion and metastasis (Menck et al., 2015). However, EV‐associated glycoproteins may exhibit atypical localisations (Sok Hwee Cheow et al., 2015), and more thorough investigations are needed to determine their surface accessibility and clinical utility for targeted EV‐based liquid biopsies.

### 3.4 Other post translationally modified EV surface proteins

Protein phosphorylation modifications are also frequently investigated in EVs. However, there are limited large‐scale studies and most reports have assessed phosphorylation in EVs *en masse*, rather than selectively assessing EV surface modifications. Protein phosphorylation is a major regulatory mechanism for signalling pathways and plays a pivotal role in protein structure, function and localisation (Zhang et al., 2018). Stable protein phosphorylation is typically limited intracellularly (Yalak & Vogel, 2012) and at present, no reliable diagnostic extracellular phosphoprotein biomarkers have been identified (Chen et al., 2017). Extracellular proteins are phosphorylated through various mechanisms, including intracellular phosphorylation before protein secretion, or extracellular phosphorylation by soluble kinases or membrane‐bound kinases (Yalak & Vogel, 2012). Phosphoproteomic analyses of human urinary‐EVs were first attempted in 2009, where phosphorylation sites were identified in membrane proteins, such as G‐protein‐coupled receptors and mucin‐1 (Gonzales et al., 2009). More recently, human plasma‐EVs were identified to carry around 7000 unique phosphopeptides, with 1934 and 479 phosphoproteins identified in large and small‐EVs, respectively (Chen et al., 2017), and functionally annotated to cancer metastasis, membrane reorganisation and intercellular communication (Chen et al., 2017). It is possible that many of these phosphoproteins are distributed at the EV surface, yet further investigations are required to deduce the EV surface phosphoproteome.

Redox modifications have also been reported for EV surface‐associated protein thiols. Different protein classes such as antibodies, receptors, hormones and enzymes often contain cysteine residues with thiol groups that undergo reversible modifications in response to changes in the redox environment (Trivedi et al., 2009). Thiol groups of cysteine residues maintain disulphide bonds, which are essential for correct protein three‐dimensional structure and function. Reversible oxidative modifications of thiols through highly reactive compounds such as reactive oxygen species, reactive nitrogen species and reactive carbonyl species, disrupt disulphide bonding and affect protein conformation, functionality and signalling (Benedikter et al., 2018). Interestingly, thiols at the EV surface are important for facilitating interactions with proteins that bear reactive thiol moieties in the extracellular environment (Santucci et al., 2019). In the blood, EVs are generally associated with redox‐active proteins that function in coagulation and immune regulation. EVs associated with oxidised thiols can promote coagulation with tissue factor (TF) and initiate the extrinsic coagulation cascade (Benedikter et al., 2018). Similarly, EV surface associations with the albuminome (Tóth et al., 2021) may take place via thiol interactions (Turell et al., 2013), extending the half‐life of EVs in the circulation (Buzás et al., 2018). EV surface thiols may also play important roles promoting or limiting inflammatory conditions. In autoimmunity, EVs carrying thiol‐dependent redox enzyme, peroxiredoxin 1, are elevated and serve as markers of inflammation (Szabó‐Taylor et al., 2017). However, under conditions of pro‐inflammatory oxidative stress, monocytes secrete EVs with fewer exofacial thiols and thiol‐dependent redox enzymes (i.e. peroxiredoxin 1) in an enzymatically inactive form (Szabó‐Taylor et al., 2017). As oxidised EV proteins form molecular patterns that trigger further inflammation, a reduction of EV surface thiols may offer systemic protection from high levels of inflammation (Binder et al., 2016).

Unfortunately, high‐throughput analyses of EV surface protein PTMs remain a challenge, as proteins can simultaneously display multiple PTMs (Palmisano et al., 2012). At the EV surface, it is likely that PTMs are unstable under different environmental conditions and during EV sample processing, leading to inefficient characterisations (Putz et al., 2012). Among the various PTMs that decorate the EV surface, protein glycosylation has been the most studied using advanced mass spectrometry (Saraswat et al., 2015; Zaia, 2010; Zhang et al., 2020) and microarrays functionalised with lectins and carbohydrate‐binding proteins (Williams et al., 2018). While PTMs display aberrant expression in various pathologies and may hold significant clinical utility in association with EVs (Kailemia et al., 2017), a detailed overview of the PTMs that adorn the EV surface is not yet available.

### 3.5 Nucleic acids at the EV surface

There is a growing body of evidence showing that the EV surface carries nucleic acids that play significant roles in intercellular communication. Chromosomal DNA has been reported to be pre‐dominately found on the surface of outer membrane vesicles shed by bacteria (Bitto et al., 2017). At the surface, this chromosomal DNA is postulated to mediate horizontal transfer of genes into eukaryotic cells for antibiotic resistance, stress response, virulence and metabolism (Bitto et al., 2017). In mammals, multiple DNA binding proteins have been identified on EVs (Table 1 and Figure 2) suggesting that stable DNA sequences may associate with the EV surface (Németh et al., 2017; Xu et al., 2019). Indeed, chromosomal (GAPDH and p53) and mitochondrial DNA (mitochondrial control gene (mt) and 12S RNA (RNR1)) fragments have been reported at the EV surface (Table 1) (Fischer et al., 2016; Németh et al., 2017). In a mast cell study, the majority of EV‐associated DNA localised to the EV surface; whether DNA associates to the EV surface during biogenesis or after secretion is unknown (Shelke et al., 2016). EV surface‐associated DNA has been shown to contribute to the net negative charge and aggregation of EVs (Shelke et al., 2016), and can be taken up by recipient cells in a time‐dependent manner (Fischer et al., 2016; Shelke et al., 2016), facilitate horizontal gene transfer (Fischer et al., 2016), and bind to ECM components, such as fibronectin (Németh et al., 2017). Interestingly, EV surface DNA has been linked to autoimmune conditions such as systemic lupus erythematosus (SLE) (Sisirak et al., 2016). SLE is characterised by DNAas1L3 deficiency, which is usually produced by dendritic cells and macrophages, and digests chromatin associated to the surface of circulating EVs. A loss of this mechanism due to DNAas1L3 deficiency in SLE, leads to the accumulation of EV surface chromatin that then acts as a self‐antigen and induces the formation of autoantibodies (Sisirak et al., 2016).

Compared to DNA, RNA species on the EV surface have been less extensively studied. Yet, the EV surface is decorated with an abundance of RNA binding proteins (RBP) and RNA nucleoproteins (RNPs) (Table 1) (Xu et al., 2019). In a study by Xu et al., miRNA let‐7a‐5p was identified at the EV surface, protected from enzymatic degradation by its association with RBPs/RNPs (Xu et al., 2019). As it is common for EV RNA profiling studies to remove extravesicular RNA contaminants by RNase digestion, it is presumed that surface RNAs are regularly overlooked in EV RNA profiling studies, which may explain the lack of reported RNA species at the EV surface (Xu et al., 2019). However, the possibility of co‐isolating RBP‐RNA complexes with EV preparations that contribute to the surface molecular repertoire cannot be discounted. It was recently observed that most extracellular RNA is associated with non‐vesicular supermeres (<50 nm) that may co‐isolate with EV preparations. Supermeres are enriched in specific miRNA species such as miR‐1246, as well as miRNA‐binding proteins AGO1, AGO2, hnRNPA2B1 and XPO5 (Zhang et al., 2021).

## 4 THE EV SURFACE INTERACTOME

The EV surface coronal layer carries an assortment of biologically active molecules that play important roles in modulating the extracellular environment. Through a diverse range of molecular interactions, the EV coronal layer governs EV mobility to local or distant sites, docking on recipient cell membranes, interactions with the ECM or effector molecules and the activation of signalling cascades in recipient cells. The vast array of interacting elements at the EV surface includes ECM molecules, cytokines, lipoproteins, immunoglobulins and all their respective receptors, as well as nucleic acids complexed with DNA‐ and RNA‐binding proteins (Figure 1). Generally, it is assumed that EVs elicit functional changes in recipient cells through membrane fusion (Hutcheson & Aikawa, 2018; Schorey & Harding, 2016; Sierro & Grau, 2019) however, the EV surface has an interesting capacity to directly target and elicit functional changes in recipient cells without membrane fusion, suggesting that cell entry may not be an obligatory process for EV signalling (Gross et al., 2012). For example, Wnt proteins at the EV surface activate Wnt signalling in HEK293 recipient cells (Gross et al., 2012). In principle, there are multiple modes of EV‐target cell interactions involving tetraspanins, integrins, ECM proteins, immunoglobulin superfamily members, proteoglycans and lectins (French et al., 2017; Mulcahy et al., 2014), however at present, these diverse interactive mechanisms between EV and cell surfaces are not well understood.

EVs are heavily decorated with ECM molecules, such as integrins and heparin sulphate proteoglycans, that facilitate cell motility; EV surface‐associated α4β1 integrin binds to fibronectin in the ECM (Rieu et al., 2000) and EV surface heparan sulphate proteoglycans facilitate their cellular uptake (Purushothaman et al., 2016). In tumours, EV uptake allows the recycling and redistribution of fibronectin and integrins to the tumour cell surface where they mediate tumour cell motility by facilitating adhesion and detachment with the ECM (Purushothaman et al., 2016; Sung et al., 2015). EV surface molecules also confer multi‐drug resistance by expressing membrane multi‐drug efflux transporters, such as P‐glycoprotein (ABCB1/MDR1/P‐gp) (Bebawy et al., 2009; Jaiswal et al., 2012) and multidrug resistance‐associated protein 1 (ABCC1/MRP1) (Goler‐Baron & Assaraf, 2011); P‐gp transfers a multi‐drug resistance phenotype after EV uptake by recipient cancer cells (Bebawy et al., 2009; Jaiswal et al., 2012). Also in cancer, numerous EV surface molecules trigger immunosuppression, including FasL, TNF, TGF‐β, CD39 and CD73 (Czernek & Duchler, 2017), by activating immune suppressor cells, modulating antigen presentation, and inducing T‐cell apoptosis (Czernek & Duchler, 2017; Pelissier Vatter et al., 2021). Programmed death‐ligand 1 (PD‐L1) on the surface of tumour‐EVs can suppress CD8^+^ T‐cell function and allow tumour expansion (Chen et al., 2018), while surface complement proteins skew B cells and monocytes towards an anti‐inflammatory phenotype (Koppler et al., 2006). The EV surface also modulates the immunological synapse between T cells and antigen‐presenting cells (Gutierrez‐Vazquez et al., 2013; Mittelbrunn et al., 2011); endothelial EVs carry antigen presentation molecules (e.g. foreign antigens, ICAM‐1, VCAM‐1, CD40, MHC I, MHC II), that form conjugates with both CD4+ and CD8+ T cells (Leone et al., 2018; Lindenbergh & Stoorvogel, 2018; Wheway et al., 2013; Wheway et al., 2014). Interestingly, exosomes from thymic epithelial cells present self‐antigens to effector memory and regulatory T cells, contributing to the lineage development of thymocytes (Skogberg et al., 2013; Skogberg et al., 2015).

In the circulation, the EV surface interacts with autoantigens, immunoglobulins, complement factors and coagulants. This interactome contributes to a more diverse coronal molecular repertoire that expands EV functionality and influences EV hydrodynamic size and mobility. In a study by Tóth et al., EVs incubated with platelet‐free and EV‐depleted plasma acquired an extended protein coat at the EV surface (Table 1), that contributed to a higher EV floatation density (Tóth et al., 2021). Here, nine plasma proteins were confidently identified as contributors to the EV coronal network, ApoA1, ApoB, ApoC3, ApoE, complement factors 3 and 4B, fibrinogen α‐chain, immunoglobulin heavy constant γ2 and γ4 chains (Tóth et al., 2021). EVs also associate with albumin and other soluble proteins, lipids and peptide hormones in the circulation that contribute to the ‘albuminome’ (Gundry et al., 2007; Holewinski et al., 2013; Tóth et al., 2021). Circulating EVs also carry complement proteins C3b and C5b‐9, and complement regulatory proteins C1‐INH, CD55 and CD59, that stimulate complement activation (Yin et al., 2008).

In autoimmune conditions, circulating‐EVs associate with autoantibodies and form pro‐inflammatory immune complexes that contribute to pathology (Buzas et al., 2014). For example, synovial fluid EVs from rheumatoid arthritis patients display the autoantigens, vimentin and fibrinogen, that form highly pro‐inflammatory immune complexes, and activate neutrophils to perpetuate inflammation (Cloutier et al., 2013). In SLE (Winberg et al., 2017) and renal disease (Karpman et al., 2017), EVs in the circulation exhibit altered surface binding with higher C3d and lower C3b/C3ib, which may contribute to chronic inflammation. Compared to normal physiology, the surface of circulatory‐EVs derived from thrombotic or oncogenic states encompass procoagulant features that initiate the coagulation cascade, such as externalised anionic PS and transmembrane TF (Owens & Mackman, 2011), that facilitate electrostatic interactions with positively charged coagulation cascade proteins (factor VII, IX, X and prothrombin) (Zwicker et al., 2009). Interestingly, platelet‐EVs are enriched with procoagulant features at their surface, which enhances coagulation by approximately 50–100‐fold compared to platelets (Sinauridze et al., 2007). Interestingly, exosomes in the circulation bind to soluble proteins at a much greater extent than microparticles, which is likely influenced by their relatively large surface area to volume ratio, EV surface properties (charge and molecular binding partners) and the composition of the biological medium (Santucci et al., 2019). Improving our understanding of how the EV surface interactome changes as a consequence to, or indeed because of, pathological processes or EV biodistribution, is vital for the development of clinically useful liquid biopsies. Further, resolving specific surface‐accessible EV markers will theoretically allow the targeted capture of specific EV populations from body fluids for more precise diagnostic and surveillance liquid biopsy strategies.

## 5 MECHANICAL AND HYDRODYNAMIC PROPERTIES OF THE EV MEMBRANE

To elicit functional changes in the local and systemic environment, it is essential for EVs to migrate from their cell‐of‐origin to recipient cells. The capacity for EV mobility through the extracellular environment is greatly influenced by EV mechanical properties and hydrodynamic size (Skliar et al., 2018). The mechanical features of the EV membrane, namely membrane elasticity, stiffness and curvature (LeClaire et al., 2021), are determined by the lipid and protein composition of a membrane (Dimova, 2014; Graham & Kozlov, 2010; McMahon & Gallop, 2005; Rawicz et al., 2000; Sorkin et al., 2018; Vorselen et al., 2020; Yi et al., 2011). The membrane curvature relates to the physical bending of membranes to generate enclosed membranous compartments, while mechanical stiffness is the resistance of the EV membrane to deformation, and elasticity of an EV membrane allows it to return to its original state after exposure to stress (LeClaire et al., 2021). The hydrodynamic diameter of an EV particle reflects how an EV particle diffuses when in solution. The hydrodynamic diameter depends on the diameter of the particle core (membrane‐enclosed EV particle), as well as the thickness of surrounding coronal layer and the concentration and type of ions in the medium (Maguire et al., 2018). These biophysical properties of EVs offer important insights into EV biology, heterogeneity, vesiculation and biodistribution patterns (LeClaire et al., 2021).

The EV hydrodynamic diameter is generally measured by nanoparticle tracking analysis (NTA) (Margolis & Sadovsky, 2019) and dynamic light scattering (DLS) methods (Varga et al., 2020). Such techniques correlate the Brownian motion of an EV particle in solution with its size (Varga et al., 2020), which offers the benefit of rapid EV size measurements. There are, however, limitations to EV size measurements using DLS and NTA. DLS measures the intensity of scattered light from particles with a bias towards larger‐EVs that scatter more light. This means that DLS is suitable only for monodisperse EV samples. NTA allows more accurate size measurements of polydisperse EV samples as it measures individual particle diffusion, however this method is still dependent on light scattering, which contributes to an overestimation of average measured EV particle sizes (Hou et al., 2018; Röding et al., 2011). In addition, NTA and DLS are highly sensitive to the temperature and viscosity of the EV medium, which may result in EV aggregation/dissociation and affect the precision of their size measurements (Jose et al., 2019). Atomic force microscopy has been useful for measuring EV membrane structure, mechanics and biomolecular features as it exerts small forces at the EV surface with a probe without causing damage to the sample (LeClaire et al., 2021; Vorselen et al., 2020). The EV membrane‐envelope can also be assessed by cryo‐TEM imaging (Zabeo et al., 2017) however, the molecular coronal layer cannot be resolved by cryo‐TEM due to its low excess density (Skliar et al., 2018).

The ability to generate high‐curvature membrane compartments is essential for eukaryotic life, allowing for constant turnover of membranes by vesicle trafficking (McMahon & Boucrot, 2015). Multiple molecular features within the lipid bilayer contribute to membrane curvature, namely lipid shape and protein motif insertion (McMahon & Gallop, 2005). Within the EV membrane, cylindrical lipids, PC and PS, form monolayers. Conical lipids that have a small head group, such as PE and PA, impose a positive curvature to a lipid monolayer, allowing it to bend in a manner that directs the phospholipid headgroups to cluster closer together (McMahon & Boucrot, 2015). Inverted conical lipids, such as PI, have a larger headgroup and confer a negative curvature, causing the phospholipid headgroups of the lipid monolayer to bend away from each other (McMahon & Boucrot, 2015). Mass spectrometric analyses of exosome membranes have identified multiple positive curvature promoting free fatty acids along with lysophosphatidyl derivatives, as well as negative curvature promoters such as cardiolipin and transmembrane proteins, for example, ion channels, transporters and receptors, that contribute to membrane bending depending on their intrinsic shape (conical or inverted conical) (Haraszti et al., 2016). In addition, high local concentrations of proteins that bind to the membrane surface have also been suggested to induce membrane curvature by a crowding mechanism (Stachowiak et al., 2012), although the extent of their contribution is still unclear (McMahon & Boucrot, 2015).

In addition to the membrane‐associated proteins and phospholipids, membrane curvature and shape is further stabilised by the cytoskeletal structure of the enclosed membrane compartment, as well as the scaffolding structure that coats the outer membrane (McMahon & Gallop, 2005). High‐resolution imaging shows that EV populations exhibit morphological heterogeneity, as a result of filamentous scaffolding structures within the EV lumen (Zabeo et al., 2017). The majority of EV populations (>80%) are comprised of single, spherical vesicles, while a small proportion of EVs is non‐spherical (Zabeo et al., 2017); tubular EVs carry filaments parallel to the length of the tubule, while rounded EVs have filaments arranged in parallel or randomly distributed.

EV membranes require mechanical stability to prevent permanent deformation during biological processes that subject EVs to mechanical stress, such as distribution through the peripheral circulation. Understanding EV membrane bending is important for the prediction of EV cellular uptake (Banquy et al., 2009), mobility (Vorselen et al., 2020), circulation time and biodistribution patterns (Anselmo et al., 2015; Skliar et al., 2018), as well as the physiological role, and vesiculation events that produce EVs of diverse configurations and sizes (Sorkin et al., 2018; van Dommelen et al., 2012; Vorselen et al., 2018; Yue & Zhang, 2013). For example, atomic force microscopy assessments of EVs from patients with hereditary spherocytosis show that they display significantly softer membranes and distinct membrane proteins compared to healthy individuals, which is postulated to be associated with higher vesiculation rates in patients (Vorselen et al., 2018). Generally, membrane stiffness, or bending rigidity, is affected by protein organisation, rather than lipids, in the EV membrane (Laulagnier et al., 2004). Membranes of higher protein–lipid ratios exhibit reduced stiffness (Fowler et al., 2016; Sorkin et al., 2018; Vorselen et al., 2018). Generally, proteins that are integral to the EV membrane significantly influence membrane rigidity, while peripheral proteins have no effect (Fowler et al., 2016). EV membranes also display varying stiffness in distinct environmental conditions, determined using fluorescent membrane probes (Laulagnier et al., 2004). In average human physiological conditions (i.e. 37°C, pH 7), EV membranes are more rigid than their respective parental cell plasma membranes (Laulagnier et al., 2004). Under more acidic conditions, EV membrane rigidity is reduced and is more comparable to that of the parental cell membrane (Laulagnier et al., 2004).

While the mechanical properties of the EV membrane can predict the size and configuration of the EV membrane envelope, the EV hydrodynamic diameter is far more dynamic. The EV hydrodynamic diameter is the measure of EV resistance to migration, which is inversely proportional to their diffusivity. The EV diffusivity is dependent on the thickness of the surrounding molecular coronal layer, which changes in the circulation with time and spatial distribution (Skliar et al., 2018). Generally, secreted EV populations have small and uniform membrane envelopes, and exhibit broad distributions in their hydrodynamic diameters (Skliar et al., 2018). This broad distribution in EV hydrodynamic diameters within an EV population is due to the varying thickness of the EV coronal layer, which reflects the heterogenous and highly dynamic molecular composition of the EV surface that is influenced by both EV biogenesis and biodistribution (Skliar et al., 2018).

The hydrodynamic diameter of an EV particle significantly influences how EVs move through extracellular spaces and bodily fluids. EVs with larger hydrodynamic diameters exhibit greater transport resistance due to steric hindrance, while EVs with smaller hydrodynamic diameters exhibit higher mobility rates and capacity to overcome transport resistance (Skliar et al., 2018). Indeed, smaller particles (less than 500 nm) are more favourable for intravenous drug delivery as they can circulate and persist in the blood, and target pathological tissues more effectively than larger particles (Anselmo et al., 2015). Large heavily glycosylated proteins entangled at the EV surface significantly contribute to the thickness of the coronal layer (Kesimer & Gupta, 2015). EV surface proteome diversity has a substantial impact on EV steric hindrance and migration by influencing EV size, conformation, charge and peripheral interactions with recipient cells and the microenvironment (Kesimer & Gupta, 2015; Skliar et al., 2018). Nonetheless, the dynamism of the EV surface allows for steric hindrance to be overcome during EV migration by conformational flexing of the coronal layer and EV membrane (Skliar et al., 2018). For example, EV interactions with ECM proteases contributes to ectodomain shedding of EV surface proteins, thereby enhancing EV activity and mobility through the ECM (Hakulinen et al., 2004; Rupp et al., 2011).

As described above, highly abundant soluble proteins in the blood interact with the surface of circulating‐EVs and contribute to the EV coronal molecular repertoire. Collectively, these molecules increase EV hydrodynamic size, floatation density and steric resistance, which has multiple implications for the development of targeted liquid biopsies. In the blood, interactions with highly abundant serum proteins may contribute to larger‐EV sizes, higher steric hindrance, lower mobility and migration into and out of the circulation, and therefore, a longer EV half‐life. A heavily dense molecular coronal layer may also obstruct access to targetable EV surface epitopes. It is possible that as EVs traverse different body compartments, the varying conditions (i.e. pH, viscosity, and molecular composition) may contribute to molecular changes at the EV surface coronal layer. Moreover, the conditions of the surrounding medium may affect the electrostatic interactions of transiently interacting molecules at the EV surface and promote their dissociation or contact. For example, as EVs move from the circulation into the urinary system where there is a lower native pH, viscosity and molecular complexity, soluble blood molecules at the EV surface may be shed and urinary molecules acquired. As the EV biological medium may influence the composition and thickness of the EV coronal layer (Fitzgerald et al., 2018; Kesimer et al., 2009), the nature of the biological medium and its effect on the EV surface should be considered when developing methods for targeted capture of disease‐specific EV surface molecules. A more thorough understanding of the EV surface and the changing molecular surface repertoire related to their systemic distribution is particularly important in the context of developing EV liquid biopsies. Indeed, assessing different body fluids to determine optimal capture of specific EV populations may expedite the development of targeted liquid biopsies.

## 6 EV SURFACE PROPERTIES AND COLLOIDAL STABILITY

The ability to successfully utilise biological nanoparticles such as EVs for liquid biopsies is highly dependent on inter‐particle interactions between EVs and the EV particle medium. Indeed, interactions between nanoparticles in dispersed systems are complex, and much of this complexity is attributed to a particulate's surface charge (Midekessa et al., 2020), the electrostatic double layer, as well as other intermolecular and surface forces (e.g. van der waals forces, steric repulsive forces, hydration forces, and specific attractive forces such as depletion and hydrophobic forces) (Moore et al., 2015). Generally, a colloidal system of EVs is stable when there are strong repulsive forces between EV particles that minimise their aggregation and deposition. Generally, EV colloidal stability is maintained by the surrounding EV electrical double layer that counteracts van der Waals forces present between proximate EV particles. Stable colloidal systems of EVs are particularly important for ensuring successful, reproducible and robust downstream assays, as aggregated EVs can skew cellular uptake and signalling, interfere with EV surface profiling, fluorescence signalling, as well as impede the targeted capture of specific EV populations from heterogenous body fluids for liquid biopsies (Midekessa et al., 2020; Williams et al., 2019).

EVs carry a surface charge in aqueous environments due to ionisation/dissociation of surface groups, and due to surface adsorption of charged molecules or ions (Midekessa et al., 2020; Moore et al., 2015). The net surface charge of EVs is commonly characterised by the zeta potential, which is the electrostatic potential of the particles measured at the shear plane, that is, at the distance from the surface where ions are not bound to the particle (Moore et al., 2015). The zeta potential offers an assessment of the collective behaviour of nanoparticles and determines their tendency towards aggregation, as the surface charge has significant influence on the stability of particle–particle and particle–medium interactions (Midekessa et al., 2020). A stable colloidal system of EVs is reflected by negative zeta potential values of a magnitude of 30–40 mV. Various physiochemical factors can influence EV surface charge and zeta potential, ranging from EV surface chemistry and the pH and ionic strength of the dispersing medium. These physiochemical factors affect ionisation of the EV surface groups, protonated states, inter‐ and intramolecular bonding, H‐bonding and ion adsorption from electrolytes present in the solution (Midekessa et al., 2020). For example, EVs suspended in higher salt buffer concentrations have higher zeta potentials (i.e. less negative zeta potential) due to higher ion conductivity, which destabilises the electrical double layer surrounding the EV membrane, reduces the repulsive forces between EV particulates and causes a collapse in the colloidal system by promoting EV aggregation due to van der Waals forces (Midekessa et al., 2020). However, high ion concentrations do not always result in the collapse of colloidal systems, showing that steric repulsion may also play important roles in maintaining the colloidal stability of EVs (Midekessa et al., 2020).

EVs also display significantly higher zeta potentials and a greater propensity towards aggregation in acidic conditions, and in dispersing mediums that contain ions of higher valency (Al^3+^/Ca^2+^) as compared to monovalent ions (Na^+^/K^+^) (Midekessa et al., 2020). This is because the EV surface harbours abundant anionic sites that can be neutralised by protons (H^+^) under acidic conditions, or interact with ions of higher valency, that lead to the compression of the surrounding electrical double layer and a higher zeta potential (Midekessa et al., 2020). As the dispersing medium significantly influences the colloidal stability of EVs, it is likely that EVs from different body fluids and cell types differ in zeta potential (Williams et al., 2019). Hence, it is proposed that any conclusions drawn from a study of the zeta potential of an EV sample may only be valid with that specific type of EV sample used (Williams et al., 2019). Furthermore, it is imperative to understand the effect of the physiological fluid in which the EVs are suspended on EV colloidal stability as heterogenous physiological fluids, such as blood, urine, cell culture medium and PBS have varying ionic strengths and macromolecules that adsorb onto the EV surface and influence the hydrodynamic behaviour and colloidal stability of EVs (Moore et al., 2015).

A uniform coating of charged macromolecules or proteins on the EV surface improves colloidal stability as the surface macromolecules offer a shield to nanoparticle interactions, as well as through combined electrostatic and steric repulsion forces. Interestingly, the molecular composition of the surrounding EV coronal layer significantly affects an EV particle's colloidal stability. Under physiological conditions, the EV surface typically carries a negatively charged network of heavily glycosylated proteins intercalated within the membrane bilayer that contribute to stability through electrostatic and repulsion forces (Kesimer et al., 2009). EVs from airway epithelial cells carry large, filamentous and entangled mucins, that are enriched with 2,6 sialic acid residues at the terminal positions (Kesimer & Gupta, 2015). At physiological pH, sialic acid residues at the terminal positions of glycoproteins and glycolipids, have an ionised carboxylate that imparts a net negative charge to the EV surface (Kesimer & Gupta, 2015). Glycosidase treatments of the EV surface alter EV electrostatic potential towards neutrality, showing that glycoproteins contribute to the net negative surface charge of EVs and ensure a stable colloidal system (Williams et al., 2019). Membrane lipids also contribute to the net negative surface charge of EVs as non‐ionic detergents that interfere with lipid–lipid and lipid–protein interactions confer higher zeta potentials to EVs (Midekessa et al., 2020). Furthermore, the hydrophilic nature of the EV surface facilitates peripheral interactions with soluble proteins, which influences EV surface electrostatic charges (Santucci et al., 2019).

## 7 EMERGING EV SURFACE TARGETED CAPTURE AND PROFILING TECHNOLOGIES FOR LIQUID BIOPSIES

In clinical molecular biology, EVs hold significant promise for biomarker discovery and the design of robust EV‐based non‐invasive liquid biopsies is a key objective for a multitude of pathologies (Hallal et al., 2019; Liu et al., 2021; Marca & Fierabracci, 2017; Morrison & Goldkorn, 2018). Various disease states have been characterised by altered EV secretion patterns, where disease‐related EVs are reported to be released in significantly greater quantities into the peripheral blood (Eldh et al., 2014; Freeman et al., 2018), where they are stable (Lai et al., 2014; Rank et al., 2011), accessible (Li et al., 2013; Skog et al., 2008; Thakur et al., 2014) and carry an array of potential disease‐specific biomarkers (Osti et al., 2019). Strategies that can selectively target EV surface molecules, and reproducibly capture and isolate specific EV sub‐populations from complex biofluids will be a watershed development for clinical molecular diagnostics. At present, microfluidic‐ and microarray‐based approaches are under investigation for their capacity to utilise the biophysical and biochemical properties of the EV surface for EV isolation, detection and characterisation (Li et al., 2017).

Microfluidic technologies control the flow of EV suspensions within microchannels and serve as robust multifunctional systems that allow for direct EV isolation and analysis, while reducing sample volume and processing time. Microfluidic systems take advantage of the natural surface charge of EVs and utilise ion‐selective membranes to trap and saturate EVs that flow onto the surface for characterisation (Cheung et al., 2018). To enhance EV capture and analysis, microfluidic chips with integrated immunoaffinity capture (He et al., 2014), acoustic‐based nanofiltration (Lee et al., 2015), pressure or electrophoretic‐driven nanoporous membrane filtration (Davies et al., 2012), nanoscale deterministic lateral displacement (Smith et al., 2018), electrokinetics (Ibsen et al., 2017; Lewis et al., 2018) and centrifugation (Sunkara et al., 2019) are under development. These systems aim to isolate and assess EVs from whole biological fluids, which could reveal avenues for developing inexpensive, portable and rapid EV‐based diagnostic systems using only a small starting sample volume (Davies et al., 2012). However, this technology is still in the early stage of development and is currently disadvantaged by low throughputs, relatively long processing times, complicated manual operation, mixing and clogging issues and compromised EV quality and purity (Pang et al., 2020).

Generally, immuno‐based microfluidic devices are the most common microfluidic technologies used for capturing and isolating EV sub‐populations. These microfluidic devices utilise fluorescent antibodies or affibodies coated on the surface of a chip to capture EV surface antigens and have been adapted to allow for ‘on chip’ molecular profiling, which holds significant clinical potential as a portable diagnostic system (Doyle & Wang, 2019; He et al., 2014). Electrokinetic approaches have also been widely integrated into immuno‐based EV microfluidic systems and are used for EV characterisation by measuring changes to the streaming current upon EV surface binding to an antibody‐functionalised microcapillary (Cavallaro et al., 2019). This technique does not require pre‐dilution of the EV sample or intermediate labelling, regardless of sample viscosity, thereby allowing EV detection from small sample volumes, and reproducible semiquantitative expression of EV surface markers (Cavallaro et al., 2019). In addition, electrokinetic methods can provide valuable information about EV size, as changes to the streaming current is strongly dependent on EV size (e.g. larger EV particles induce stronger changes in the streaming current) (Cavallaro et al., 2019). Electrokinetic methods have been employed for label‐free EV detection in pancreatic and brain cancer patient blood (Ibsen et al., 2017; Lewis et al., 2018), and have shown significant capacity to quantitate changes to EV surface protein expression during tumour progression and treatment resistance.

Antibody microarray platforms also show promise for EV surface phenotyping (Belov et al., 2016). Microarrays interact with EV surface proteins for targeted capture and isolation of specific EV populations by targeting their most frequently expressed surface proteins and assessing expression levels by fluorescent or chemiluminescent signals. More recently, antibody microarray platforms have been modified to assess antibody‐captured vesicles in label‐free modes using SPR imaging (SPRi) (Rojalin et al., 2019) and single particle interferometric reflectance imaging sensor (SP‐IRIS) (Daaboul et al., 2016). However, targeting EV surface proteins still presents several drawbacks: (1) the EV surface proteome and topology is not yet well‐defined, (2) surface profiles are affected by soluble antigens peripherally associated to the EV surface, (3) inherent variability in antibody specificity and affinity, and (4) fluctuations in the relative abundance of protein markers (Gori et al., 2020).

Generally, immuno‐based microfluidic and microarray technologies have utilised tetraspanins (CD9, CD81 and CD63) and tumour‐cell surface associated markers such as EpCAM, CA125, CD19, EGFR, EGFRvIII, podoplanin and PDGFR for on‐chip capture of tumour‐EV sub‐populations (Belov et al., 2016; Cavallaro et al., 2019; Daaboul et al., 2016; He et al., 2014; Reategui et al., 2018). A sub‐population of glioblastoma‐EVs were isolated from the peripheral blood by immunoaffinity targeting of the oncogenic variant protein, EGFR/EGFRvIII, which showed 94% tumour‐EV specificity (Reategui et al., 2018), while microarrays functionalised with antibodies to colorectal cancer cell surface proteins allowed the capture of colorectal cancer LIM1215‐EVs owing to a 70.6% (24/34) overlap in LIM1215 cell surface antigen expression (Belov et al., 2016). These immunocapture studies have improved our understanding of the EV surface and the capacity for targeted EV capture from heterogenous body fluids. Further rigorous investigation to define panels of surface EV markers will enhance the targeted capture of specific tumour‐EV sub‐populations from heterogenous clinical samples.

Membrane‐sensing peptides have also emerged as conceivable molecular probes that can be immobilised on chips for targeting the EV lipid membrane and capturing EVs (Gori et al., 2020). Small‐EVs are characterised by their highly curved membranes, with outer leaflets containing high levels of anionic phospholipids and lipid‐packing defects. Membrane‐sensing peptides can sense and selectively bind to curved membranes (Lemmon, 2008; Zeno et al., 2019), form strong electrostatic and hydrophobic interactions with phospholipids in the outer membrane leaflet, and insert into membrane defects (Zeno et al., 2019). Unfortunately, membrane‐sensing peptides are difficult to remove from the EV surface as they form strong interactions with the EV membrane and require strong dissociation conditions that can affect EV membrane integrity. On the other hand, membrane‐penetrating peptides that are rich in lysine and arginine residues and have an affinity for negatively charged phospholipid bilayers of EVs, offer the advantage of reversible binding and gentle dissociation from EVs (Ishida et al., 2020; Nakai et al., 2016).

As EV surface molecular arrangements are not yet adequately characterised, more recent adaptations have been made to microfluidic technologies to completely omit antibodies and other molecular probes from the capture and analysis of EVs (Jalali et al., 2021). Recently, a nanobowtie fluidic device was developed that allows for probe‐free capture and label‐free molecular profiling of EVs. The microfluidic device utilises surface enhanced raman spectroscopy (SERS) to provide molecular fingerprints of captured EVs for their lipid, nucleic acid, cholesterol and protein content, and C = C vibrations (Jalali et al., 2021). This device was adapted with plasmonic nanostructures embedded at the bottom of the fluidic chamber to capture EVs and used an enhanced electromagnetic field to uniformly distribute EVs into a monolayer to enhance SERS detection sensitivity of EV‐associated molecules. This device could generate a library of SERS spectra for EV molecular cargo and by observing pattern differences in SERS spectra was capable of distinguishing between cancer‐derived and non‐cancer‐derived EVs, and synthetic liposomes (Jalali et al., 2021).

Several other methods are also under development for probe‐free capture of EVs from body fluids, offering the possibility of targeting universal EV surface properties for liquid biopsies (Gori et al., 2020). Integrated nanoscale deterministic lateral displacement arrays isolate EVs by size through parallel pillar arrays on a single chip that fractionate small EVs with zigzag modes and larger EVs with bump modes (Smith et al., 2018). Centrifugal microfluidic devices can also be used to enrich for EVs from urine, cell‐culture (Woo et al., 2017), complete blood and plasma (Sunkara et al., 2019); EVs are enriched within 30 min at a relatively low g‐force (less than 500 × g) and use sequential and tangential flow‐filtration, which involves two nanofilters with pore diameters of 600 and 20 nm, to isolate significantly higher EV yields.

EV surface profiling through microfluidic and microarray techniques will be highly valuable for assessing panels of EV surface markers. However, in‐depth biomarker discoveries are first required to construct the EV surface molecular profile (Wu et al., 2019). Unfortunately, while a range of methods has been developed for EV isolation and characterisation, these methods remain time‐consuming and expensive (Cavallaro et al., 2019). Further refinement of these methods will help overcome the requirements for large sample volumes, extensive labelling, signal amplification or sample enrichment, and is anticipated to improve the ability to detect specific EV populations in complex biofluids (Cavallaro et al., 2019).

## 8 CONCLUSION

The EV surface carries a plethora of molecules that are integral and peripherally associated to the EV membrane that interact with soluble molecules in the extracellular environment, influencing EV size, mobility and biodistribution patterns. Together, EV surface molecules form a coronal layer that surrounds the EV membrane and contributes to a large, interactive and highly dynamic surface area that facilitates functionally important interactions and downstream signalling events. In the context of clinical molecular diagnostics, EV surface molecules are critically important for the development of liquid biopsies that can target and capture disease‐specific EV populations from complex body fluids. The expansion of EV surface studies to construct a comprehensive multi‐dimensional spatial and temporal molecular map of the EV surface is warranted and will be important to further our understanding of the roles of EVs in health and disease, and to develop robust EV‐based assays for clinical molecular biology.

## AUTHOR CONTRIBUTIONS

*Conceptualization*: Susannah Hallal and Kimberley L. Alexander. *Investigation*: Susannah Hallal, Ágota Tűzesi and Kimberley L. Alexander. *Visualization*: Susannah Hallal and Kimberley L. Alexander. *Writing – original draft*: Susannah Hallal, Ágota Tűzesi, Michael E. Buckland and Kimberley L. Alexander. *Writing – review & editing*: Susannah Hallal, Ágota Tűzesi, Michael E. Buckland and Kimberley L. Alexander. *Resources*: Michael E. Buckland and Kimberley L. Alexander. *Supervision*: Kimberley L. Alexander. *Funding acquisition*: Kimberley L. Alexander. *Project administration*: Kimberley L. Alexander.
